# Spin Trapping: A Review for the Study of Obesity Related Oxidative Stress and Na^+^/K^+^-ATPase

**DOI:** 10.4172/2155-9899.1000505

**Published:** 2017-05-17

**Authors:** Athar Nawab, Alexandra Nichols, Rebecca Klug, Joseph I. Shapiro, Komal Sodhi

**Affiliations:** 1 Department of Medicine, Joan C. Edwards School of Medicine, Marshall University, USA; 2 Department of Surgery and Biomedical Sciences, Marshall University, USA

**Keywords:** ROS, Oxidative stress, Na^+^/K^+^-ATPase, Spin trap

## Abstract

Reactive oxygen species (ROS) have gained attention with mounting evidence of their importance in cell signaling and various disease states. ROS is produced continuously as a natural by-product of normal oxygen metabolism. However, high levels ROS causes oxidative stress and damage to biomolecules. This results in loss of protein function, DNA cleavage, lipid peroxidation, or ultimately cell injury or death. Obesity has become a worldwide epidemic; studies show fat accumulation is associated with increased ROS and oxidative stress. Evidence exists supporting oxidative stress as a factor driving forward insulin resistance (IR), potentially resulting in diabetes. Na^+^/K^+^-ATPase signaling is also a potential source of ROS promoting oxidative stress. The best way to observe radical species in biological systems is electron paramagnetic resonance spectroscopy with spin trapping. EPR spin trapping is an important technique to study the mechanisms driving disease states attributed to ROS.

## Introduction

Processes increasing the formation of radical oxygen species contribute, at least in part, to the induction of numerous diseases. An imbalance between ROS production and antioxidants guides a system into oxidative stress. Numerous studies have found an association between obesity and increased production of radical species exists in the development of atherosclerosis, diabetes, and hepatic disease, among others. Several studies which explore interventions to decrease ROS production has shown to halt the development of metabolically related pathology. To further elucidate the impact of ROS on organ dysfunction, methods of quantifying ROS prove useful. Accurate and sensitive quantification of ROS is challenging, but EPR with spin trapping demonstrates a reliable method [1]. Involving EPR spin trapping in the investigation of experimental treatments aids in the delineation of key mediators driving ROS production, oxidative stress and disease.

## Reactive Oxygen Species and Oxidative Stress

In healthy aerobes, the production of ROS is roughly balanced with antioxidant defense systems. The term oxidative stress was coined to describe conditions of increased intracellular ROS levels and reflects an imbalance between radical production and antioxidant defenses. It can be defined as, “a disturbance in the pro-oxidant–antioxidant balance in favor of the former, leading to potential damage,” [2]. This insult is called ‘oxidative damage’. ROS are produced naturally as intermediates in many biological redox reactions such as the synthesis of water, when O_2_ molecules pair with elemental hydrogen to form H_2_O.

**Figure 1:**
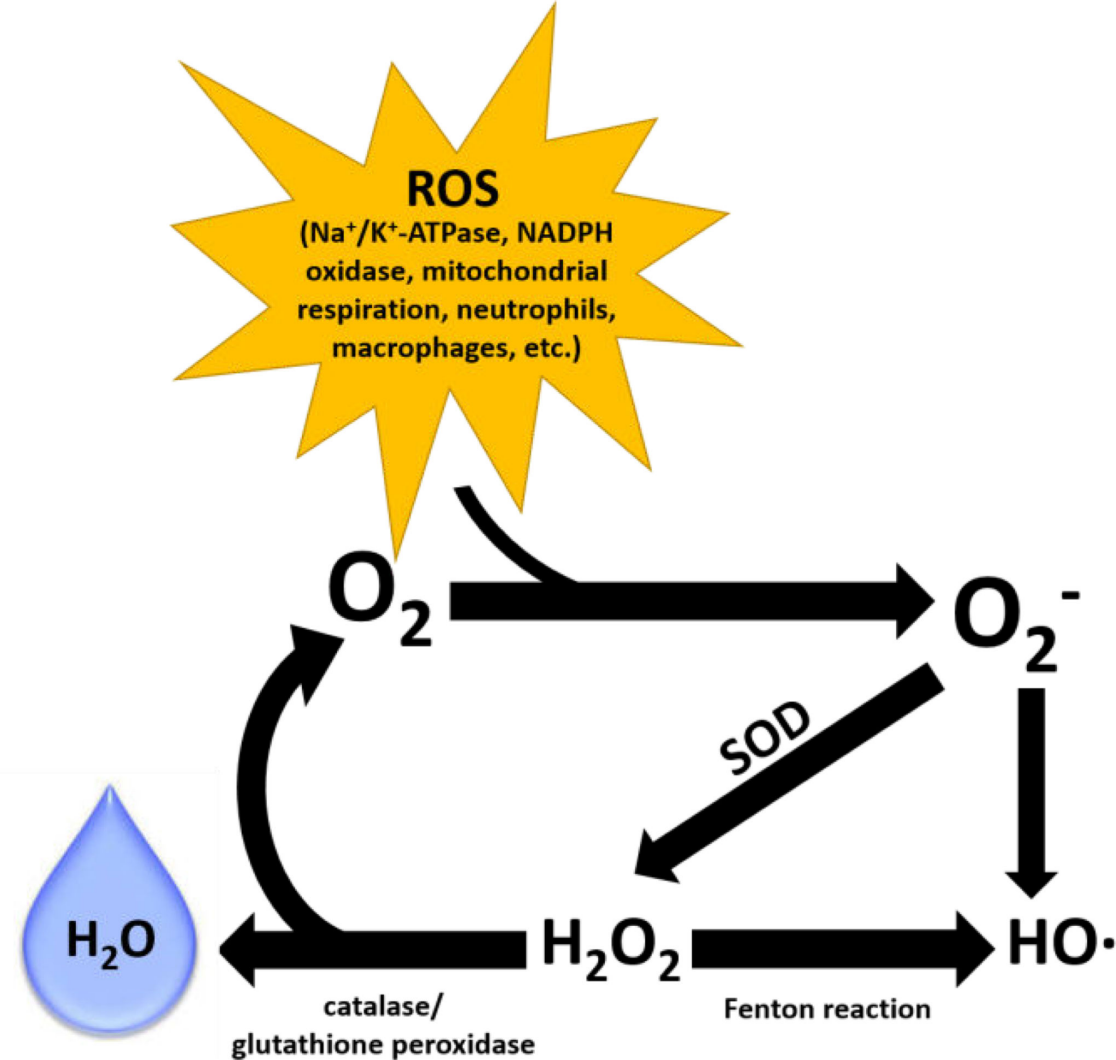
Chemistry of ROS. Reactions involved in the production and removal of oxygen free radicals in the cell. Oxygen is initially converted to superoxide (O_2_^−^) by a cellular enzyme. O_2_^−^ is converted to hydrogen peroxide by superoxide dismutase (SOD). Hydrogen peroxide is converted to water by either catalase or glutathione peroxidase. In an alternative scenario, a Fenton reaction converts hydrogen peroxide into hydroxyl ion and hydroxyl radical.

ROS are highly reactive chemical entities comprised of two major groups: free radicals (e.g., O_2_^−^, hydroxyl [OH^−^], nitric oxide [^·^NO]), and non-radical derivatives of O_2_ (e.g., H_2_O_2_, ONOO^−^). Free radicals are any species capable of independent existence, containing one or more unpaired electrons. This unpaired electron imparts immense reactivity, making the molecule highly unstable. Non-radical derivatives are less reactive and have a longer half-life than free radicals (Figure 1) [3].

Many endogenous and exogenous processes can generate radicals in biological systems. They can be formed as part of enzyme catalytic cycles and have beneficial properties, i.e., defense mechanisms, and signaling molecules [4]. Additionally, endogenous ROS formation is a consequence of the electron transport chain; which causes electron leakage and generation of O_2_^−^ radical anion. Dismutation of this species yields hydrogen peroxide (H_2_O_2_). H_2_O_2_ can be used by peroxidase enzymes to generate radicals either deliberately or unintentionally [5]. Reactions with trace levels of redox-active transition metal ions like iron or copper are another well-established source of radicals. Other internal sources include redox-active enzymes used to oxidize substrates to radicals and activated white cells that use oxidative chemistry to tackle bacterial parasitic and fungal infections. In these cases, NADPH oxidases serves as a major source of O_2_^−^ radical. Acute and chronic inflammation are now well-established sources of oxidative damage in many human diseases (e.g. heart disease, rheumatoid arthritis, asthma, cystic fibrosis, and significant number of cancers) [6].

External radicals are a major source of oxidant stress to biological systems. These include various types of high-energy radiation, atmospheric pollutants, solvents, among other chemical free radicals in biology and medicine. Cellular damage incurred by the biochemical effects of ROS arises from lipid peroxidation, protein and DNA modification. At low concentrations, ROS also has an important role in activating signal transduction. ROS is produced continuously as natural by-products of oxygen metabolism leading to oxidative damage, loss of protein function, DNA cleavage, lipid peroxidation, or cellular injury and death [7].

## ROS in Obesity and Diabetes

With the global population of obese individuals (BMI ≥ 30) estimated at approximately 400 million, the risk of metabolic syndrome, diabetes, and cardiovascular-related mortality are an increasing concern [8]. There is a well-established correlation between obesity and ROS production. Studies have shown that fat accumulation in obese individuals is positively correlated with systemic oxidative stress in both humans and mice [9]. Mouse models of obesity have shown that the production of ROS increases selectively in the white adipose tissue (WAT) of obese mice, suggesting that increased oxidative stress is due to increased ROS production from accumulated fat [9]. In order for proper cellular function, a baseline level of ROS is required. Additionally, in isolated situations, ROS does improve IR. However, chronic exposure to ROS is detrimental to normal physiological conditions [10,11].

Of clinical concern, excessive ROS production is one of many factors with a possible role in the development of IR^−^ a central feature of type 2 diabetes. There are two types of indirect evidence for this: (1) the strong correlation of markers for oxidative stress with the presence of obesity and diabetes, and (2) experiments showing that direct treatment of 3T3-L1 adipocytes with high doses of hydrogen peroxide or the accumulation of other ROS-inducing agents can promote IR [12,13]. Pharmacological and genetic interventions designed to decrease ROS levels significantly prevented the development of IR, insinuating that ROS plays a causal role in the development of IR [12].

Many studies suggest the production of ROS is increased in diabetes [13–16]. Chronic exposure of β-cells to a high glucose concentration causes impairment of insulin biosynthesis and secretion through a process called β-*cell glucose toxicity*. In a diabetic state, hyperglycemia and subsequent production of ROS decrease insulin gene expression and initiate apoptosis [17,18]. ROS production is enhanced by hyperglycemia which increases the formation of glycation products, altered polyol pathway activity, and increased O_2_^−^ release from mitochondria [19–22]. Pancreatic-cell dysfunction and IR are hallmarks of diabetes mellitus. It has been shown that ROS is involved in both the progression of IR as well as pancreatic-cell dysfunction [23,24]. Suppression of ROS in obese type 2 diabetic mice restores both cell function and insulin sensitivity and amelioration of glucose intolerance. It is likely that ROS is closely associated with the development of type 2 diabetes and related complications [25]. Redox imbalance is central to pathophysiology of chronic disorders, including obesity, metabolic syndrome, and diabetes. These disorders are associated with a feed-forward loop of oxidative stress, eventually leading to end organ damage. Additionally, in non-diabetic human subjects, adipose accumulation is correlated with markers of systemic oxidative stress and studies suggest that systemic oxidative stress positively correlates with BMI [26,27].

Oxidative stress is known to impair both insulin secretion by pancreatic β cell, glucose transport in muscle and adipose tissue [28–30]. Increased oxidative stress in vascular walls is involved in the pathogenesis of hypertension and atherosclerosis [31,32]. Oxidative stress underlies the pathophysiology of hepatic steatosis [33]. Studies suggest that increased ROS secretion into the circulation is also involved in induction of IR in skeletal muscle and adipose tissue, impaired insulin secretion by β cells, atherosclerosis and hypertension [34]. Increased oxidative stress in accumulated adipose *via* increased NADPH oxidase and decreased antioxidant enzymes [35]. This causes dysregulated local production of adipokines, or adipocytokines, including: plasminogen activator inhibitor-1 (PAI-1), tumor necrosis factor-alpha (TNF-α), resistin, leptin, IL-6 and adiponectin. PAI-1, adiponectin and leptin dysregulation alters energy expenditure, decreases insulin sensitivity and increases inflammation. Release of IL-6 into the circulation bolsters IR and carbohydrate intolerance. Increased levels of leptin and TNF-α causes proliferation and dysfunction of vascular endothelial cells; thereby promoting atherosclerosis. Increased circulating resistin, due to obesity, stimulates a state of hyperglycemia [35]. Increased ROS production from accumulated adipose leads to increased oxidative stress in blood, this hazardously affecting other organs including the liver, skeletal muscle, and aorta. Thus, increased oxidative stress in accumulated fat is an early instigator and one of the important underlying causes of obesity-associated metabolic syndrome [9].

## Spin Trapping/Electronic Paramagnetic Resonance (EPR) Spectroscopy

The ideal assay for ROS detection should have sufficient sensitivity to ensure that measurements made are within the linear range of the assay and significantly above the limits of detection. Current assays focus on detection of specific radical species such as O_2_^−^. These rely on cytochrome c reduction, chemiluminescence from lucigenin and related dyes, nitro blue tetrazolium reduction to formazan, fluorogenic oxidation of hydroethidine to ethidium, or aconitase inactivation/activation. Nearly all of these assays have been criticized for various reasons [36–38]. For example, in the evaluation hydroethidine oxidation to ethidium, the conversion to ethidine may be useful as a qualitative indicator of O_2_^−^. It should not be used as a quantitative measure of a radical since it catalyzes the dismutation of O_2_^−^ and therefore the yields of ethidine per O2− are less than stoichiometrically equivalent [37]. There should be specificity for radical species at physiological and pathophysiological concentrations. Additionally, it should be applicable to a wide variety of experimental conditions and comparable between these applications.

In high amounts, reactive nitrogen species cause nitrosative stress and act with ROS to damage cells. NO is an important signaling molecule and a critical regulator of various cellular functions. There is evidence that NO has cytotoxic effects due to reaction with another free radical, O_2_^−^, the superoxide anion that produces peroxynitrite (ONOO^−^). Peroxynitrite interacts with lipids, DNA, and proteins *via* direct oxidative reactions or *via* indirect, radical-mediated mechanisms. These reactions trigger cellular responses ranging from subtle modulations of cell signaling to overwhelming oxidative injury; committing cells to necrosis or apoptosis [39]. Using spin trapping, NO has been detected in mitochondria, and mitrochondrial homogenates with great success [40]. There are several NO traps such as the complexes of iron with dithiocarbamate ligands which allow NO detection in both polar and non-polar environments [41].

Most biologically relevant radicals have very short half-lives making them impossible to detect in biological samples [42]. Because of this, compounds that form stable adducts with radicals are used. Using EPR spectroscopy, specific spectra are observed for the radical in question. Using various spin traps in conjunction with ESR, it is possible to identify and quantify different families of radical species in a manner not possible with other assays [43]. With selection of the proper probe, the measurements are restricted to intracellular or extracellular compartments [41]. The cost, knowledge of operation and upkeep of this large, complicated piece of equipment is a drawback to this type of investigation [44]. However, having expertise in using an EPR spectrometer gives the potential of allowing specific measurements of a diverse array of radical species that could not otherwise be detected by other assays.

Few reliable methods exist for detecting free radicals in biological systems. Electron paramagnetic resonance (EPR) spectroscopy is often described as the “gold standard” for detection and characterization of oxygen-derived free radicals in biological systems *via* the spin trapping method [45]. Since EPR makes it possible to study materials with unpaired electrons, it can provide a wealth of information appropriate to the study of radical species. The basic concepts of EPR are analogous to those of nuclear magnetic resonance; this involves the absorption of microwave energy by paramagnetic species produced during transition of spin states in the presence of an external magnetic field [46].

EPR spectroscopy can detect and identify radical species, such as O_2_^−^ and hydroxyl. With the development of spin trapping methods, it is possible to accurately detect and identify short-lived free radical species undetectable under normal conditions. Spin trapping involves a reaction in which the radical is combined to a spin trap molecule forming a spin adduct; this moiety is then detected by EPR spectroscopy [47]. Radicals such as the O_2_^−^ anion react with spin traps moieties such as: nitrone and nitroso, or metal complexes. A covalent bond is formed from the reaction of nitrones and nitroso with reactive radicals, and a coordination bond is formed between NO and Fe^2+^ complexes. The resulting EPR spectrum exhibits a hyperfine splitting pattern that is characteristic of the trapped radical [43]. With distinctive spectra, one can identify and analyze properties of radicals such as: the mechanism, the kinetics, and the dose dependence of radical production in biological systems [41].

**Figure 2:**
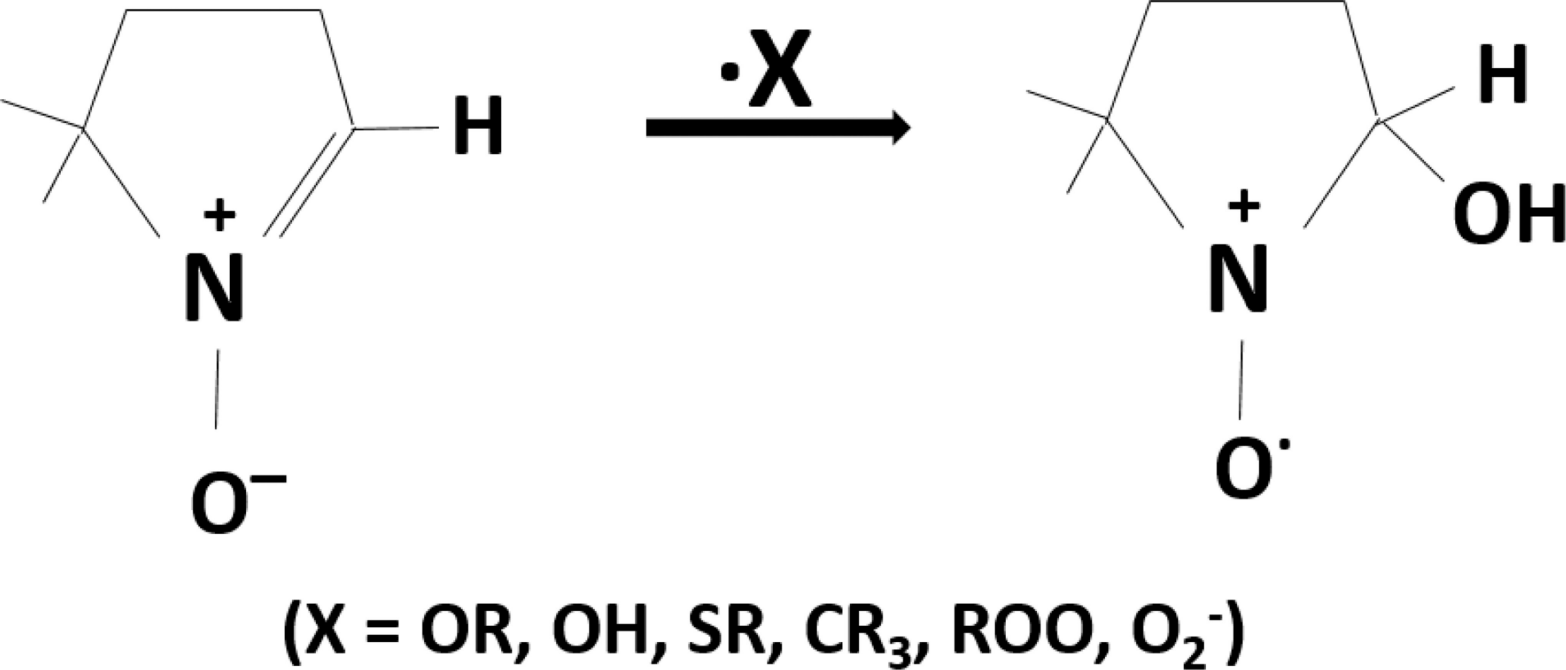
Structure of DEPMPO, 5-(diethoxyphosphoryl)-5-methyl-1-pyrroline N-oxide, spin trap.

Though the technique has been around for 50 years, application of this technique is relatively new in the study of functioning tissues, cells and live animals. The principle problem, in addition to sensitivity has been the stability of the spin adducts. The spin adducts of DMPO are traditionally unstable in cells [48]. New spin traps, such as nitrone 5-diethoxyphosphoryl-5-methyl-1-pyrroline N-oxide (DEPMPO), have greatly overcome this limitation (Figure 2). The spin trap was developed to increase the half-life of the O_2_ adduct to 14 minutes. Spin trapping is highly sensitive, capable of detecting radicals at concentrations ranging from nM to µM [47]. The DEPMPO is an example of a relatively new spin trap molecule. DEPMPO is a good reagent to use in the detection of intracellular free radicals, since it rapidly diffuses across lipid membranes [49]. DEPMPO is a phosphorylated spin trap that reacts with thiyl radicals to produce EPR spectra from two diastereomers. The phosphorus hyperfine coupling of the DEMPMO/^•^RS adduct gives very complex EPR spectral patterns, but the spectral features used to distinguish between thiyl and hydroxyl radical adducts are distinct [50]. New spin traps such as DEPMPO have overcome the problem of poor spin stability. Additionally, DEPMPO is a cyclic nitrone with low cellular toxicity, making it suitable for application in biological systems. Cyclic nitrones are highly sensitive, such that radical adducts are detected with great sensitivity and specificity even in cells [51]. Complimented to an EPR study, a wealth of information is made available about the nature of the spin trapped species. EPR is the only method to allow specific and sensitive detection of radical intermediates. There has been rapid progress in EPR spectroscopy over the last decade and it is likely to remain the standard for detecting and identifying radicals in biological systems.

Many pathological conditions are associated with ROS-mediated events [25]. While the data linking all of these processes with ROS is sometimes controversial, there is no question that ROS have an important association with many pathophysiological and physiological processes. Since progress has been made in the study of these mechanisms, there is now an increased need to develop and utilize methods capable of measuring reactive species more sensitively and quantitatively.

## Na^+^/K^+^-ATPase

Na^+^/K^+^-ATPase has been extensively studied for its functioning as an ion pump. In the past several decades, it has also been discovered to function as a scaffolding and signaling protein. It was observed that cardiotonic steroids (CTS) mediate signal transduction through the Na^+^/K^+^-ATPase and result in the generation of reactive oxygen species (ROS). Reactive oxygen species are also capable of initiating the signal cascade. In recent years, this Na^+^/K^+^-ATPase/ROS amplification loop has demonstrated significance in oxidative stress related disease states, including obesity, diabetes, and atherosclerosis [52]. The discovery of this novel oxidative stress signaling pathway holds significant therapeutic potential for the aforementioned conditions, as well as others that are rooted in ROS.

The Na^+^/K^+^-ATPase enzyme (“sodium pump”) was discovered to have scaffolding and signaling function by Dr. Zijian Xie in the late 1990s. Our work with obesity models has provides evidence that the Na^+^/K^+^-ATPase signaling cascade worsens obesity, diabetes, and dyslipidemia, as these conditions are all related to an imbalance of oxidative stress. Xie’s model of Na^+^/K^+^-ATPase signaling arose from difficulties explaining signaling with the ionic model along with experimental observations of reactive oxygen species and tyrosine kinase activities being critical to such signaling. The model proposes that the Na^+^/K^+^-ATPase α-1 subunit serves as a negative regulator of Src. During conformational changes in α1 induced by CTS or oxidation, Src becomes active and triggers a signal cascade, which involves the generation of ROS. The α-1 subunit of the Na^+^/K^+^-ATPase binds Src and maintains Src in an inactive state. However, CTS binding appears to alter the Na^+^/K^+^-ATPase structure allowing Src to become activated. In turn, this trans-activates the EGFR and begins the signal cascade, which causes an increase in ROS, activating additional Na^+^/K^+^-ATPase molecules, as well as causing downstream activation of ERK and effects nuclear transcription. Blockade of Na^+^/K^+^-ATPase-induced ROS amplification might attenuate the development of disease states [52–59].

**Figure 3:**
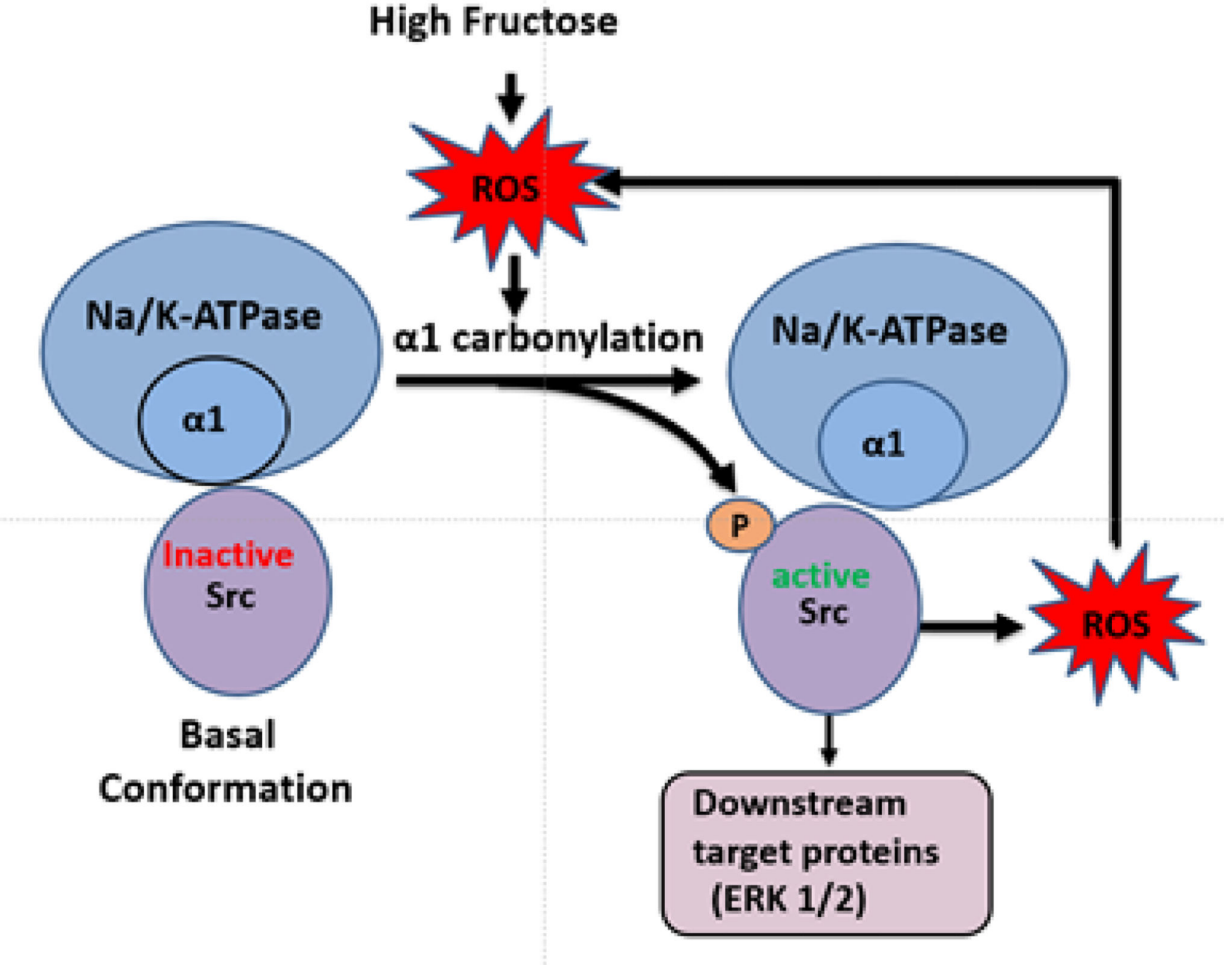
Schematic diagram of the Na/K ATPase-ROS-Src pathway.

Atherosclerosis is perpetuated by oxidative stress and chronic inflammation. Studies indicate that ROS causes direct injury to vascular cells and encourages the accumulation of oxidized LDL [34]. Oxidative stress stimulates Na/K-ATPase/Src/ROS amplification loop tempering c-Src and ERK1/2 signaling which results in dyslipidemia (Figure 3) [34]. Adipocytes that accumulate during the state of obesity are responsible for increased ROS and systemic oxidative stress. Amplification of oxidant signaling is shown to occur *via* the feed-forward Na/K-ATPase-Src-EGFR signaling loop during adipogenesis [34]. Non-alcoholic steatohepatitis (NASH) emerges from complex pathophysiology initiated by oxidative stress and inflammation. Obesity and IR are responsible for the amplification of the Na/K-ATPase/ROS pathway contributing to NASH. Increased circulating levels of free-fatty acids and triglycerides, generate ROS, this perpetuating oxidative stress to initiate the Na/K-ATPase/ROS amplification pathway [56]. Further hepatocellular injury results from direct damage caused by inflammatory cytokines. With the increased production of ROS, the Na/K-ATPase signal cascade is responsible for the proliferation of kupffer cells and upregulation of collagen expression leading to the formation of fibrosis [56].

## Conclusion

Reactive oxygen species have received much attention recently for their role in driving forward various disease processes. ROS generation increases the oxidative stress in a system and a well-established link has been found between increased ROS, diseases including obesity, diabetes, atherosclerosis, and chronic kidney disease. Studies investigating interventions capable of reducing the formation of radical species have been observed to prevent pathologies such as IR from developing in mice that were experimented on. Na^+^/K^+^-ATPase signaling is another way oxidative stress can accumulate in cells as it is also a pathway that results in the production of radical species. The most reliable way to observe radicals is using EPR spin trapping. By using this technique, the rate of free radical production can be determined for distinct radical species such as the superoxide and the hydroxyl radicals. Information like this may prove very useful in determining the mechanisms that drive ROS production and how they bring about the pathologies of the different disease states they are indicated to be increased in.
